# Occupational exposure, carcinogenic and non-carcinogenic risk assessment of formaldehyde in the pathology labs of hospitals in Iran

**DOI:** 10.1038/s41598-024-62133-9

**Published:** 2024-05-25

**Authors:** Parvin Foroughi, Farideh Golbabaei, Mohsen Sadeghi-Yarandi, Mehdi Yaseri, Mahta Fooladi, Saba Kalantary

**Affiliations:** 1 Department of Occupational Health Engineering, School of Public Health, Tehran University of Medical Sciences, Tehran, Iran; 2 Department of Epidemiology and Biostatistics, School of Public Health, Tehran University of Medical Sciences, Tehran, Iran; 3 Department of Occupational Health and Environment, Iran Mineral Processing Research Center, Tehran, Iran

**Keywords:** Carcinogenic, Non-carcinogenic, Formaldehyde, Occupational exposure, Pathology lab, Risk, Health care, Health occupations

## Abstract

Formaldehyde, a known carcinogenic compound, is commonly used in various medical settings. The objective of this study was to assess the carcinogenic and non-carcinogenic risks associated with occupational exposure to formaldehyde. This study was conducted in the pathology labs of four hospitals in Tehran. Cancer and non-cancer risks were evaluated using the quantitative risk assessment method proposed by the United States environmental protection agency (USEPA), along with its provided database known as the integrated risk information system (IRIS). Respiratory symptoms were assessed using the American thoracic society (ATS) questionnaire. The results indicated that 91.23% of exposure levels in occupational groups exceed the NIOSH standard of 0.016 ppm. Regarding carcinogenic risk, 41.03% of all the studied subjects were in the definite carcinogenic risk range (LCR > 10^−4^), 23.08% were in the possible carcinogenic risk range (10^−5^ < LCR < 10^−4^), and 35.90% were in the negligible risk range (LCR < 10^−6^). The highest index of occupational carcinogenesis was observed in the group of lab technicians with a risk number of 3.7 × 10^−4^, followed by pathologists with a risk number of 1.7 × 10^−4^. Furthermore, 23.08% of the studied subjects were within the permitted health risk range (HQ < 1.0), while 76.92% were within the unhealthy risk range (HQ > 1.0). Overall, the findings revealed significantly higher carcinogenic and non-carcinogenic risks among lab technicians and pathologists. Therefore, it is imperative to implement control measures across various hospital departments to mitigate occupational formaldehyde exposure levels proactively. These findings can be valuable for policymakers in the health sector, aiding in the elimination or reduction of airborne formaldehyde exposure in work environments.

## Introduction

Formaldehyde is a colorless gas with a pungent and irritating smell, produced on a large scale through the oxidation of methanol gas^1^. Formaldehyde is used as a stabilizing agent for pathology samples and in embalming, as well as a tissue preservative and disinfectant in anatomy, pathology, and microbiology labs^2,3^. In 2017, the production of this substance was reported to be approximately 52 million tons^4^. The earnings generated from the global utilization of industrial-grade formaldehyde are projected to experience an annual growth of 3.77% between 2017 and 2022. Approximately 700,000 workers in North America are believed to be directly engaged in industries where formaldehyde is utilized. In contrast, within the European Union, the number of workers exposed to formaldehyde above the background levels is estimated to be 1.7 million, with 175,380 of them located in Italy^5^.

Exposure to formaldehyde can result in irritation of the eyes and upper respiratory tract, as well as an increased risk of nasopharyngeal cancer and leukemia^6^. The food and drug administration (FDA), the consumer product safety commission (CPSC), and the environmental protection agency (EPA) have expressed serious concern about the carcinogenicity of formaldehyde^2^. The potential carcinogenic risk to humans has been studied in a number of cohort and case-control studies. Finally, this substance was classified as a definite carcinogen by the international agency for research on cancer (IARC). In epidemiological studies on industrial workers, pathologists and anatomists, the relationship between exposure to formaldehyde and an increased risk of various types of cancer including: nasal cavity, nasopharynx, lung, brain, pancreas, prostate, colon and atopic lymphoma system has been determined^7^.

Inhalation is the main route of occupational exposure to formaldehyde. The allowable threshold limit of time–weight average 8 h occupational exposure (PEL-TWA) to formaldehyde in the US occupational safety and health administration (OSHA) is 0.75 ppm. The American conference of governmental industrial hygienists (ACGIH) has recommended a ceiling limit of 0.3 ppm for the irritant effects of formaldehyde exposure on the eyes and upper respiratory tract^7^. Due to its high solubility, this compound can irritate the upper respiratory system. Upper airway irritation is the most common effect reported by exposed workers, which has occurred in some cases at low concentrations. Symptoms of upper airway irritation include dry or sore throat, itching and burning sensation in the nose, regular cough, wheezing and shortness of breath^7,8^. About 90 to 95% of formaldehyde is absorbed in the upper respiratory tract and a small amount reaches the alveoli^9^.

The findings from toxicokinetic studies suggest that the main absorption site for inhaled formaldehyde in humans is at the point of entry. The absorption site of formaldehyde from the respiratory tract is influenced by various factors, such as airway anatomy, breathing pattern, and ventilation rate. In some studies, it has been reported that the half-life of formaldehyde in the bloodstream, following absorption in the respiratory tract, is approximately 1 min. Generally, it is documented that inhaled formaldehyde undergoes rapid biotransformation^10^. Various efforts have been made to biologically monitor formaldehyde exposure, including investigating DNA–protein cross-links in individuals exposed to formaldehyde and assessing sister chromatid exchange rates in personnel exposed to formaldehyde within the range of 0.04–6.9 ppm. Additionally, formaldehyde levels were measured in the exhaled breath of cancer patients and cigarette smokers during clinical monitoring^10^. Formaldehyde is quickly oxidized to formic acid and enters biological systems, or it is excreted as water and carbon dioxide through exhalation^8^.

The results of formaldehyde measurement studies in hospitals indicate high concentration of this pollutant in different wards^8^. Zain et al. showed that hospital workers in Malaysia exhibited significantly elevated levels of formaldehyde and experienced noticeable symptoms of poor health^11^. Soltanpour et al. demonstrated in a systematic review study that significant health risks, both related to cancer and non-cancer outcomes resulting from exposure to airborne formaldehyde (such as neurological, dermal, and respiratory effects), were noteworthy in both industrial and healthcare environments^4^. A simulation study was conducted by the park et al. to investigate the concentration and assess the health risks of individuals in long-term exposure to formaldehyde following a chemical incident in Korea. Cancer risks from inhaling and non-cancer risks from the soil intake were evaluated. The elevated cancer risk remained below 1.0 × 10^−6^, suggesting that inhalation exposure to formaldehyde did not result in adverse health effects. However, the hazard index exceeded 1, affirming that exposure to soil containing formaldehyde led to detrimental health impacts^12^. Protano et al. conducted a systematic study aiming to investigate the carcinogenic effects of formaldehyde in occupational exposures. Findings indicated a limited level of evidence supporting a correlation between occupational exposure to formaldehyde and the incidence of cancer^13^. The study by Kwak et al. did not provide evidence supporting an increased risk of lung cancer in occupational exposure to formaldehyde; however, they noted that considering the substantial increase in risk estimates observed in recent high-quality studies, the potential for exposure to formaldehyde to elevate the risk of lung cancer should be taken into account^9^.

Therefore, considering the numerous effects and consequences of this combination on human health, it is crucial to assess the risks of carcinogenesis, overall health, and respiratory symptoms caused by occupational exposure to it^14^. To prevent and reduce the risks associated with chemical exposure in the workplace, risk assessment is essential^11^. Risk assessment serves as a proactive measure that is carried out to evaluate and manage unwanted incidents. It involves a detailed study of what can cause harm to people in the workplace, determining whether existing preventive measures are sufficient, or if additional measures should be taken to prevent damage^4,15^.

The national research council (NRC) defines risk assessment as the determination of potential adverse health effects of exposure to environmental hazards. The quantitative risk assessment method introduced by the US environmental protection agency (EPA) is one of the most reliable methods available in this field. Today, many international organizations, including the World Health Organization, consider the use of quantitative risk assessment methods as the basis for the legislation of chemical compounds^15^.

Therefore, given the significance of the aforementioned issues and the high exposure of healthcare workers in Iran to formaldehyde, along with the absence of such studies in the investigated hospitals despite the high exposure of individuals in their pathology departments and the insufficient control measures in these areas, there is a crucial need to prioritize control measures and raise awareness among decision-makers for their proper implementation. Consequently, this study was conducted with the aim of assessing the carcinogenic and non-carcinogenic risks and occupational health effects of formaldehyde exposure in selected hospitals in Tehran, Iran.

## Materials and methods

### Study design

This descriptive cross-sectional study was conducted in four major hospitals in Tehran, Iran, which will be referred to as hospitals A, B, C, and D. Survey were performed during the winter season of 2022. Similar exposure groups (SEGs) were employed for the surveillance of individual exposure. SEG can be described concerning a particular procedure, a specific section of the procedure (in a specific physical setting), or a set of employees facing the same substance in identical conditions. When aiming to evaluate the exposure of a considerable number of individuals, they can be grouped based on their identical exposure to the pollutant^15^. The pathology labs and three occupations within it (SEG) include pathologists, lab technicians, and housekeeping staff (N = 57) were selected for study. Inclusion criteria included non-smoking status and a minimum work experience of 12 months in pathology laboratories. All subjects with a history of respiratory diseases, common colds, and flu during the survey were excluded. All methods were carried out in accordance with relevant guidelines and regulations. Moreover, in this study, to ensure the validity and reliability of data different tools such as standardized data collection protocols (for maintain consistency and reduce measurement variability), quality control measures (implementation of quality control and assurance), and regular calibration (calibrating measurement tools) were applied in different stages of data collection, that all of which were checked during data analysis.

The study protocol was approved according to Helsinki’s international guidelines and all experimental protocols were approved by ethical guidelines of Tehran University of Medical Sciences (IR.TUMS.SPH.REC.1401. 177). Prior to the research all of participations completed consent form and a statement of informed consent was obtained from all subjects.

### Formaldehyde sampling method

Personal sampling was conducted in the breathing zone of pathologist, lab technicians, and housekeeping staff to measure the occupational exposure levels of formaldehyde in the pathology labs of the four hospitals. The method prescribed by the National Institute for Occupational Safety and Health (NIOSH 3500) was employed to monitor formaldehyde exposure^12,13^. The sensitivity of this method is considered satisfactory and reliable for detecting formaldehyde decomposition in peripheral samples due to its low detection limit of 0.5 μg and the samples’ stability for 30 days at 25 °C. Briefly, personal sampling was performed with two series of glass Midget impingers containing 20 ml of 1% sodium bisulfite solution and Polytetrafluoroethylene (PTFE) membrane filters (37 mm). Each sample was taken individually using a personal sampling pump (SKC Universal PCXR8 sample pump single kit). Prior to sampling, the personal sampling pump was calibrated at a flow rate of 1 L/min^−1^ using an electronic flow meter (DC-Lite BIOS Drycal; SKC, USA). Additionally, environmental parameters such as temperature, pressure and air humidity were measured simultaneously during sampling^13,16,17^. After sampling, the samples were quickly transferred to the laboratory inside polyethylene bottles using a cool box. To prepare the samples, the following steps were taken: first, 0.1 mL of 1% chromotropic acid and 6 ml of 98% sulfuric acid were added to each sample. Then, the samples were heated at 95 °C for 15 min. Finally, they were left at room temperature for 2–3 h^17^.

### Sample preparation and analysis method

According to NIOSH 3500 method, a calibration curve was plotted using a stock solution prepared by diluting 1 mL of 1 mg/mL formaldehyde stock solution with 100 mL of 1% sodium bisulfite solution. Next, 0.1, 0.3, 0.5, 0.7, 1.0, 1.5, and 2.0 mL of the calibration stock solution were pipetted into 25 mL flasks. Then, a 1% solution of sodium bisulfite was added to each flask to increase the volume of each working standard to 4 mL. For sample preparation, 4 mL portions were taken from each sample solution and transferred to 25 mL flasks. Subsequently, 1 mL of 1% chromotropic acid and 6 mL of 98% sulfuric acid were added to each flask. The samples were then heated to 95 °C and maintained at that temperature for 15 min. Finally, the absorbance of the sample solution was determined using a UV–VIS spectrophotometer (Yoke 1700, China) at 580 nm. The formaldehyde concentration in unidentified samples was determined by comparing the absorbance of standard samples^18^. Additionally, environmental samples were employed to determine the levels of formaldehyde concentration within the restrooms of all examined units, as well as dining rooms. To achieve this objective, sampling was conducted for 100 min at a flow rate of 1 L/min^−1^. A total of 18 environmental samples were gathered (one sample per room).

### Quality control and assurance

During this research, blank samples were gathered both during field sampling and laboratory analysis to assess contamination levels and potential errors that might occur throughout the processes of sampling, transferring, and analysis. Every sample was corrected for blank levels. The results showed that the concentration of formaldehyde in each blank sample was found to be less than one percent of the values observed in the original samples. Additionally, to plot the calibration curve, various concentrations of standard solutions were prepared in accordance with the aforementioned cases. Duplicate samples were examined, and they exhibited a variation of less than 5%. The limits of detection (LOD) and limits of quantitation (LOQ) values are in the range of 0.002 ppm and 0.006 ppm respectively.

### Assessment of occupational exposure to formaldehyde

The threshold limit values (TLVs) for formaldehyde has been established by several different organizations including NIOSH, OSHA, and ACGIH. In this study TLV-TWA by NIOSH was selected for assessment because NIOSH proposes the lowest threshold (0.06 ppm) in comparison to OSHA (0.75 ppm) and ACGIH (0.3 ppm), and opting for NIOSH enhances the accuracy of risk evaluation.

NIOSH provides threshold limit values (TLVs) based on time–weighted average standards for formaldehyde, which is 0.016 ppm. These TLVs are established using a time–weighted average (TLV-TWA), indicating that the worker’s exposure time should not exceed the TWA during an 8 h workday within a 40 h workweek. It should be noted that this value is not an absolute threshold to determine the health of employees, but rather it represents a relative limit that reduces the risk of exposure of workers to chemical agents.

1 $${\text{TLV}} - {\text{TWA}}_{i} = \frac{{{\text{C}}_{{\text{i}}} {\text{t}}_{{\text{i}}} + {\text{C}}_{{\text{i}}} {\text{t}}_{{\text{i}}} + \ldots }}{{8{\text{ h}}}}$$

where t_i_ is the period of time the worker was exposed to the concentration c_i_ (i.e. formaldehyde).

Using the formaldehyde concentration data from this study and the time-weighted average threshold limit value (TLV-TWA) for formaldehyde provided by NIOSH, we calculated an occupational exposure index (Ei) using the following equation^15,19^:

2 $${\text{E}}_{{\text{i}}} = \frac{{{\text{C}}_{{\text{i}}} }}{{{\text{ TLV}} - {\text{TWA}}_{{\text{i}}} }}$$

where Ei is the occupational exposure index (dimensionless) and Ci (mg/m^3^) represents the concentration of formaldehyde. TLV-TWA_i_ value (mg/m^3^) was provided by the NIOSH (0.016 ppm or 0.019 mg/m^3^ for formaldehyde).

Due to the TLV-TWA values being based on an 8 h workday and 5 day workweek, adjustments were necessary when the weekly working hours exceeded 40 h. These corrections were made using the Brief and Scala model. To apply the Brief and Scala model in unusual exposure scenarios, the daily or weekly reduction factor in the TLV-TWA value was calculated using the following equation^1,20,21^:

3 $${\text{TLV}} - {\text{TWA}}_{{\text{C}}} = {\text{TLV}} - {\text{TWA}}_{{\text{i}}} \times \left( {\frac{40}{{\text{h}}} \times \frac{{168 - {\text{h}}}}{128}} \right)$$

Where TLV-TWA_C_ (mg/m^3^) is the adjusted occupational exposure limit (OEL) and h shows working hours per week. Within a workplace, formaldehyde having E_i_ values exceeding 1.0 is considered to pose a potential health hazard to the workers.

### Carcinogenic risk assessment based on USEPA method

In the present study, risk assessment estimation of formaldehyde exposure among workers in the pathology section was conducted using the USEPA risk assessment method and the database of the integrated risk information system (IRIS) proposed by the EPA. In this methodology, carcinogenic risk assessment was determined using the lifetime cancer risk (LCR) index. The LCR is the determination of probability of developing cancer risk due to continues exposure to carcinogenic agents such as formaldehyde over several years of activity in the work. The LCR is assessed using the chronic daily intake (CDI) and slope factor (SF). LCR can be calculated using the following equation^17,22^:

4 $${\text{LCR}} = {\text{CDI}} \times {\text{SF}}$$

where CDI is chronic daily intake (kg. day. mg^−1^) and SF is the cancer slope factor (mg.kg^−1^.day). CDI was obtained using the following equation^21^:

5 $${\text{CDI}} = \frac{{{\text{C }} \times {\text{IR}} \times {\text{ET}} \times {\text{EF}} \times {\text{ED }}}}{{{\text{BW}} \times {\text{ALT}} \times {\text{NY}}}}$$

where C represents the concentration of formaldehyde (mg. m^−3^) in the sampling area. IR denotes the mean inhalation rate (m^3^. h^−1^), ET represents the exposure time (h. week^−1^), EF is the exposure frequency (week. year^−1^), ED is exposure duration (years), BW is the body weight (kg), ATL is the average lifetime (in years), and NY is the exposure duration in one year (day/year)^23,24^. During the present study, information related to the length of the exposure period, body weight, duration of exposure and frequency of exposure was collected through a self-designed questionnaire by the researcher.

The acceptable value of LCR, as defined by the World Health Organization (WHO), is between 10^−5^ and 10^−6^ and any value less than this^25^. A value of more than 10^−4^ represents “definite risk”, while a value between 10^−5^ and 10^−4^ is considered a “probable risk”, between 10^−6^ and 10^−5^ is categorized as a “possible risk,” and a LCR value less than 10^−6^ is deemed a “negligible risk”^21^.

### Non-carcinogenic risk assessment

The potential non-carcinogenic risk of formaldehyde was quantified using the hazard quotient (HQ) equation. HQ defined the relative significant of the exposure to a compound and is calculated by denoting the ratio of exposure level to a reference concentration (RFC). The RFC represents long-term exposure through inhalation to compounds without any adverse non-cancer health effects during a human lifetime^26^. HQ was computed using the following equation^27^:

6 $${\text{HQ}} = \frac{{{\text{EC}}}}{{{\text{RFC}}}}$$

where RFC is the inhalation reference concentration (mg. m^−3^) and EC is the concentration by inhalation (mg. m^−3^). EC obtained using the following equation^15^:

7 $${\text{EC}} = \frac{{{\text{C}} \times {\text{ET}} \times {\text{ED}} \times {\text{EF}}}}{{{\text{ATL}}}}$$

where C represents the concentration of formaldehyde (mg. m^−3^), ET is the exposure time (h. week^−1^), EF is the exposure frequency (week. year^−1^), ED is the exposure duration (years), and ATL is the average lifetime (in years). A HQ > 1 indicates the possibility of non-carcinogen health effects in humans; conversely, a HQ < 1 is interpreted as evidence of a negligible risk of adverse effects^28^. All parameters used for calculating carcinogen and non-carcinogen risks in this study are summarized in Table 1.

**Table 1:** Description of parameters required for the estimation of carcinogen and non-carcinogen risk assessment.

Parameter	Description	Value	Unit	Source
Weight of evidence	–	A	–	IRIS.USEPA
		1		IARC
SF	Slope factor	0.0455	mg·kg^−1^·day	IRIS.USEPA
C	Formaldehyde concentration	–	mg. m^−3^	TWA_8_; from sampling
IR	Inhalation rate	15.7 to 16	m^3^. h^−1^	EPA exposure factors Handbook: 2011 edition
ET	Exposure time	40	h. week^−1^	Questionnaire
EF	Exposure frequency	36	Week. year^−1^	Questionnaire
ED	Exposure duration	30	Years	Questionnaire
BW	Body weight	50–92	Kg	Questionnaire
ALT	Average lifetime	70	years	WHO, ACGIH
NY	Number of days per years	310	Day/year	Questionnaire
RFC	Inhalation reference concentration	3.94 × 10^−5^	mg. m^−3^	USEPA

### Evaluation of respiratory symptoms

In this study, thoracic society division of lung disease questionnaire (ATS—DLD-78A) was used to evaluate some respiratory symptoms. ATS is a commonly used questionnaire for identifying the respiratory symptoms. It contains questions regarding frequent and chronic respiratory symptoms including cough, phlegm, wheeze and shortness of breath^15,28^. Data were collected through face-to-face interviews.

### Statistics analysis

Ultimately, in order to analyze the data, SPSS software (IBM Corp. released 2019. IBM SPSS statistics for Windows, version 26.0. Armonk, NY: IBM Corp.) was used. Mean, standard deviation (SD), frequency and percent were used in the descriptive statistics section. Kolmogorov–Smirnov normality test and Q–Q plot were employed to assess the normal distribution of data. The results of these tests indicated that the collected data did not follow a normal distribution (*p*-value < 0.05). Therefore, non-parametric tests were utilized to analyze the data at various stages of the study. To compare the prevalence of all respiratory symptoms with exposure to formaldehyde (less than and more than NIOSH TLV-TWA) we used the Mann–Whitney U test. Furthermore, to compare the prevalence of all respiratory symptoms with cancer risk (definite, probable, and negligible risk level), and non-cancer risk (high and low risk level), we applied Chi square test. The analysis of the relationship between exposure to formaldehyde and the risk of carcinogenic and non-carcinogenic as well as, relation between carcinogenic and non-carcinogenic risk among different occupational groups was conducted using the Kruskal–Wallis test. For all tests, *p*-value less than 0.05 considered statistically significant.

### Ethics approval and consent to participate

The study protocol was approved according to Helsinki’s international guidelines and ethical guidelines of Tehran University of Medical Sciences [IR.TUMS.SPH.REC.1401. 177].

## Results

### Occupational exposure to formaldehyde

The average 8 h time–weight average (TWA-8) concentration of formaldehyde found in pathology labs and exposure index (Ei) at the hospitals under study have been demonstrated in Table 2. The results indicated that 91.23% of the exposure levels in occupational groups exceed the TLV-NIOSH. The average occupational exposure to formaldehyde among all occupant groups, including pathologist staff (0.093 ± 0.06), lab technicians (0.082 ± 0.02), and housekeeping staff (0.019 ± 0.008) was above the TLV (0.016 ppm). The average occupational exposure to formaldehyde in housekeeping staff was lower than in lab pathologists and lab technician. The average exposure index in all hospitals and individual was above 1, which is considered a potential health hazard for the staff. The lowest and highest average exposure index were observed housekeeping staff and lab technicians and pathologists, respectively.

**Table 2:** Occupational exposure to formaldehyde at studied hospitals.

Hospital and job title	Occupational exposure to formaldehyde (ppm)			
	Min	Mean ± SD	Max	Mean exposure index (Ei)
A		0.06 ± 0.26		
Pathologist	0.05		0.13	4.32
Lab technician	0.05	0.08 ± 0.49	0.2	5.45
Housekeeping staff	0.01	0.03 ± 0.014	0.05	2.90
B				
Pathologist	0.05	0.06 ± 0.014	0.07	3.60
Lab technician	0.05	0.06 ± 0.014	0.07	3.60
Housekeeping staff	0.01	0.016 ± 0.015	0.03	1.17
C				
Pathologist	0.01	0.065 ± 0.077	0.12	3.94
Lab technician	0.03	0.07 ± 0.05	0.11	4.33
Housekeeping staff	0.01	0.01 ± 0	0.01	0.63
D				
Pathologist	0.19	0.19 ± 0	0.19	11.74
Lab technician	0.02	0.12 ± 0.14	0.22	7.80
Housekeeping staff	0.02	0.02 ± 0	0.02	1.25
TLV (NIOSH)		0.016 (ppm)		

### Environmental concentration of formaldehyde

The findings from the analysis of formaldehyde environmental levels in restrooms and the dining rooms are presented in Table 3. Most hospital environments studied demonstrated concentration levels above the TLV recommended by NIOSH. An exception was the environment in HCd_1_ hospital C which presented levels below TLV for formaldehyde (0.016 ppm), according to NIOSH.

**Table 3:** Environmental concentration of formaldehyde in rest rooms and dining rooms of hospitals studied.

Unit	Concentration (ppm)
Hospital A, dining room 1 (HAd_1_)	0.32
Hospital A, dining room 2 (HAd_2_)	0.06
Hospital A, rest room 1 (HAr_1_)	0.05
Hospital A, dining room 3 (HAd_3_)	0.1
Hospital A, dining room 4 (HAd_4_)	0.11
Hospital A, dining room 5 (HAd_5_)	0.14
Hospital A, rest room 2 (HAr_2_)	0.09
Hospital A, rest room 3 (HAr_3_)	0.14
Hospital A, dining room 6 (HAd_6_)	0.12
Hospital B, dining room 1 (HBd_1_)	0.09
Hospital B, rest room1 (HBr_1_)	0.06
Hospital B, rest room 2 (HBr_2_)	0.17
Hospital C, dining room 1 (HCd_1_)	0.01
Hospital C, rest room1 (HCr_1_)	0.03
Hospital C, rest room2 (HCr_2_)	0.05
Hospital D, rest room1 (HDr_1_)	0.22
Hospital D, dining room 1 (HDd_1_)	0.13
Hospital D, rest room 2 (HDr_2_)	0.26

### Carcinogenic risk assessment of formaldehyde

The results of the evaluation of the carcinogenic risk of exposure to formaldehyde showed that 41.03% of all the studied subjects were in the definite carcinogenic risk range (LCR > 10^−4^), 23.08% of all participations were in the possible carcinogenic risk range (10^−5^ < LCR < 10^−4^) and 35.90% were in the negligible risk range (LCR < 10^−6^). The highest index of occupational carcinogenic risk in the group of lab technician is 5 × 10^−4^ in hospitals A and D. The occupational group of housekeeping staff in all of Hospitals obtained the lowest carcinogenic risk index with a value of 1 × 10^−9^. The values of chronic daily intake (CDI) and the lifetime cancer risk (LCR) according to occupational groups in different hospitals are given in Table 4.

**Table 4:** Carcinogenic risk assessment results at studied hospitals.

Hospitals and job title	Chronic daily intake (CDI) (mg. kg^**−**1^. day^**−**1^)	lifetime cancer risk (LCR) (without unit)	Carcinogenic risk level (percentage)		
			Definite	Probable	Negligible
			LCR > 10^−4^	10^−5^ < LCR < 10^−4^	LCR < 10^−6^
A					
Pathologist	8.6 × 10^−3^	2.2 × 10^−4^	62.5	37.5	0
Lab technician	1.4 × 10^−2^	5 × 10^−4^	87.5	12.5	0
Housekeeping staff	2.5 × 10^−4^	1 × 10^−9^	0	0	100
B					
Pathologist	3 × 10^−3^	1 × 10^−4^	0	100	0
Lab technician	1 × 10^−2^	4 × 10^−4^	100	0	0
Housekeeping staff	1 × 10^−6^	1 × 10^−9^	0	0	100
C					
Pathologist	4 × 10^−3^	1 × 10^−4^	50	0	50
Lab technician	5 × 10^−3^	1/5 × 10^−4^	50	0	50
Housekeeping staff	1 × 10^−6^	1 × 10^−9^	0	0	100
D					
Pathologist	3 × 10^−3^	1 × 10^−4^	0	100	
Lab technician	1/5 × 10^−5^	5 × 10^−5^	0	50	50
Housekeeping staff	1 × 10^−6^	1 × 10^−9^	0	0	100

### Non-carcinogenic risk assessment of formaldehyde

The results of the non-carcinogenic risk assessment using the hazard index (HQ) method showed that 23.08% of the studied subjects were within the permitted health risk range (HQ < 1.0) and 76.92% of the subjects were within the unhealthy risk range (HQ > 1.0). The HQ values according to the different occupational units are given in Table 5. The highest average of HQ was related to lab technician workers in hospital A with a value of 17 ± 18.48. Following this, the highest calculated HQ values were observed in the pathologists (hospital A), lab technicians (hospital B), with the values of 9.1 ± 9.33, and 10.53 ± 3.5, respectively. The lowest HQ in the present study was obtained in the workers of the housekeeping units in all of hospitals.

**Table 5:** Non-carcinogenic risk assessment results at studied hospitals.

Hospitals and job title	Hazard quotient mean (SD)	95% confidence interval for the mean		Non-carcinogenic risk level (percentage)	
				High	Low
		Upper limit	Lower limit	HQ > 1.0	HQ < 1.0
A					
Pathologist	9.1 ± 9.33	1.7	30.56	100	0
Lab technician	17 ± 18.48	2.31	49.28	100	0
Housekeeping staff	0.46 ± 0.24	0.27	0.8	0	100
B					
Pathologist	3.17 ± 0.46	2.48	3.50	100	0
Lab technician	10.53 ± 3.5	8.03	13.03	100	0
Housekeeping staff	0.05 ± 0.06	0.1	0.13	0	100
C					
Pathologist	4.88 ± 6.44	0.32	9.44	50	50
Lab technician	4.60 ± 5.6	0.64	8.67	80	20
Housekeeping staff	0.03 ± 0.00	0.3	0.3	0	100
D					
Pathologist	3.38 ± 0.04	3.8	3.86	100	0
Lab technician	1.9 ± 2.15	0.38	3.43	50	50
Housekeeping staff	0.075 ± 0.007	0.7	0.8	0	100

It was found that there is a significant difference between occupational exposure to formaldehyde and carcinogenic and non-carcinogenic risk and similarly, between the carcinogenic and non-carcinogenic risk values in the studied occupational groups (pathologists, lab technicians, and housekeeping staff) (*p*-value < 0.05).

### Assessment of respiratory symptoms

**Figure 1:**
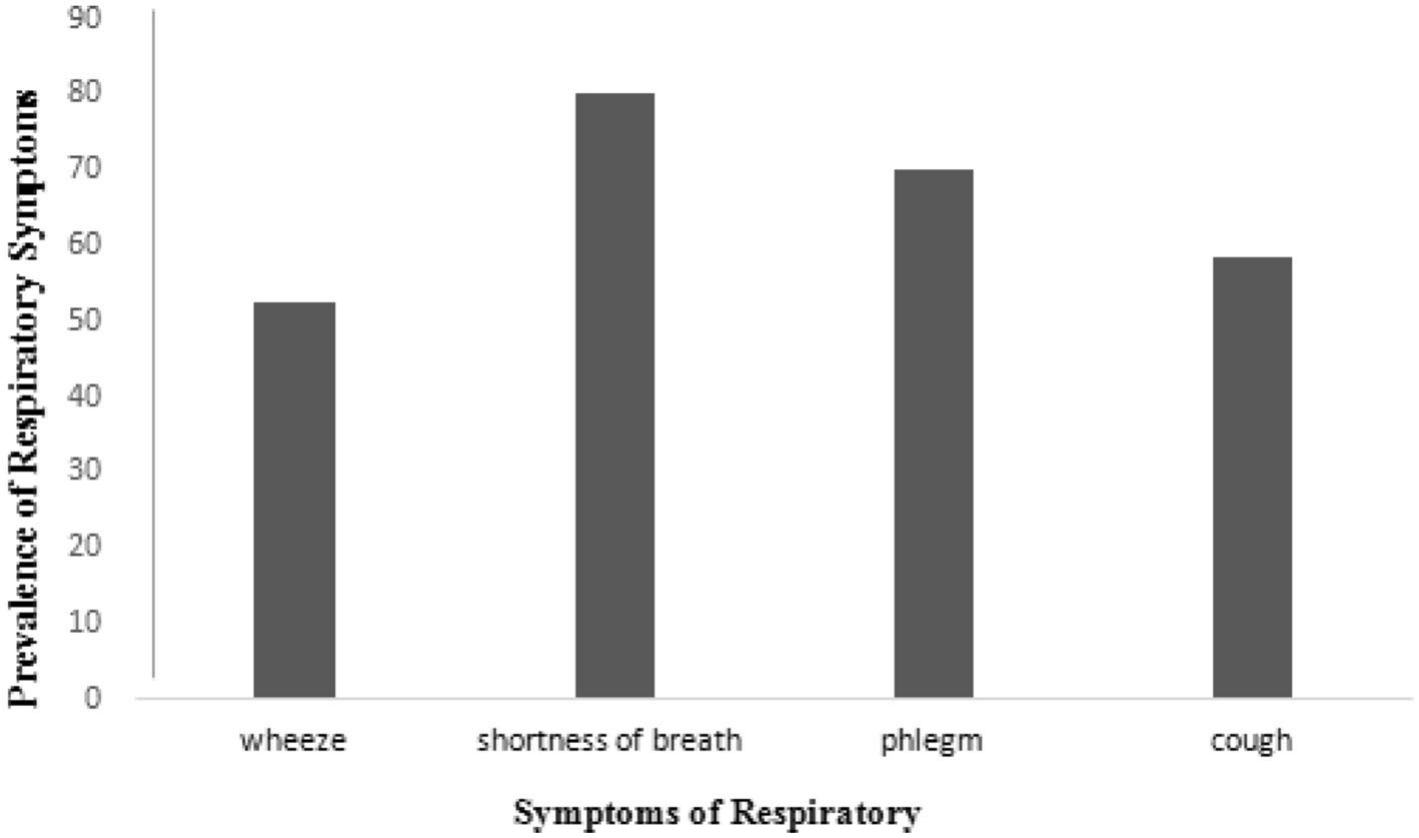
The prevalence of respiratory symptoms.

The results showed that prevalence of wheeze, cough, phlegm and shortness of breath was 52.38%, 58.39%, 70% and 80%, respectively (Fig. 1). It was found that there is a significant difference between the prevalence of all respiratory symptoms and exposure to formaldehyde (*p*-value < 0.05). However, there is no relationship between the respiratory symptoms and carcinogenic and non-carcinogenic risks (*p*-value > 0.05).

## Discussion

The purpose of this investigation was to determine the occupational exposure of staff in pathology laboratories to formaldehyde and to estimate the potential risks, both cancer and non-cancer related. The results of this study showed high exposure of many employees to formaldehyde (Table 2). The occupational exposure to formaldehyde was determined within a range of 0.01–0.22 ppm. Some investigations have been carried out in different countries regarding the occupational exposure to formaldehyde in hospital settings^29–32^. Jalali et al. (2020) reported that the exposure of pathology lab employees to formaldehyde was 0.52 ppm, above the TLV-NIOSH recommended exposure limit (0.016 ppm). The results this study indicated that 44% of lab worker and 14% of staff at the lab reception were above the exposure limit^18^. In the study of Yahyaei et al. (2020), conducted in Iran, it was found that the mean respiratory exposure of pathobiology department staff with formaldehyde in five hospitals was higher than the TLV-TWA8. Additionally, the lab technicians at hospital no. 5 had the highest weekly exposure index (0.664 ppm)^16^. This finding suggests that all the laboratory workers should receive training about working procedures to reduce the occupational exposure to formaldehyde. Furthermore, employees should avoid obstructing the ventilators on the laminar tables when handling the samples, and refrain from leaving the formalin-containing waste fluid tank unclosed^33^. As shown in Table 2, the difference in average personal exposure among occupational groups can also be attributed to difference tasks, exposure patterns, and the length of time staff are exposed. In this study, lab technicians and pathologists have higher exposure than housekeeping staff. The duties of lab technicians and pathologist including sample passage, tissue processing, modeling and cutting, and slide preparation, cause direct exposure to formaldehyde. Formaldehyde as the main compound used in sample fixation, leads to direct exposure of staff, especially lab technicians and pathologists. Due to the shorter duration and fewer presence of housekeeping staff in the laboratory compare to lab technicians and pathologists, their exposure to formaldehyde was lower. These results are in agreement with earlier studies^18,34,35^. However, these results differ from those of Assari et al. which indicated no significant difference in exposure to formaldehyde among staff of different occupational groups^8^. Nevertheless, the observed difference between studies seems reasonable given the differences in the structure of pathology laboratories in Iranian medical centers, as well as difference in the hours of staff presence in laboratory environment^18^.

In the present study, the average formaldehyde exposure in Hospital D was higher than in the other hospitals under investigation. Furthermore, the values of Ei were also higher in Hospital D compared to the other hospitals under study. This could be due to inadequate control measures, such as an improper ventilation system, improper specimen storage, and inadequate personal protective equipment. These results are in line with those of previous studies. Zian et al. found that the 8 h-time weighted average formaldehyde concentration (0.25 ± 0.11 ppm) in pathology labs exceeded the recommended limit. This research identified that formaldehyde concentration could be significantly impacted by factors such as the control measures in workplaces^11^. This was evident through the observation that almost all Hospitals exhibited higher TWA8 and STEL concentrations. This was attributed to fewer control measures in place (including a smaller grossing area, recirculating backdraft grossing station, lack of mechanical exhaust ventilation, and improper specimen storage). Therefore, it is recommended to consider employing of various control measures such as applied ducted backdraft grossing station, fume hoods, mechanical exhaust ventilator, and proper specimen storage^11^. As seen in Table 3, the environmental concentration of formaldehyde in dining and rest spaces exceeds the recommended limit. This can be attributed to the inappropriate location of these spaces, their proximity to pathology laboratories (being placed within the same laboratory space), the lack of proper partitioning and separation, low air exchange rates, inadequate ventilation, the presence of open bins containing tissues or waste contaminated with formaldehyde, and improper storage of formaldehyde in this space^11^. In addition, the personal sampling included the period that the workers spent in the dining or rest rooms, and these times were also considered in determining the amount of exposure along with the time of the work process. These findings are consistent with the study by Mota et al. (2021). In their study, environmental measurements were also taken to assess the levels of formaldehyde in the air in various environments, as well as the diffuse of formaldehyde in areas adjacent to the pathology room. Some of the measurements revealed levels exceeding the recommended limit by NIOSH^32^. This indicates attention to improving environmental conditions such as structural or technical changes in the workplace. Furthermore, in the absence of technical and structural changes, the importance of workers’ actions in limiting formaldehyde emissions becomes crucial. Workers should understand, be trained, and pay more attention to the actions they take throughout the day to reduce exposure to and emissions of formaldehyde^32^.

It can be seen from Table 4 that cancer risk associated with formaldehyde exposure ranged from 1 × 10^−^9 to 5 × 10^−^5. The obtained ranges of cancer risk are lower than the negligible cancer risks of 10^−6^ (one in 1,000,000) and higher than the define risk of 10^−4^ (one in 10,000) recommended by USEPA. Yahyaei et. al (2020) reported a similar range of formaldehyde carcinogenic risk for different units in pathology labs and occupations, ranging from 1.0852 × 10^−6^ to 4.7889 × 10^−4^, which aligns closely with the values in this study^16^. Karami Mosafer et al. presented formaldehyde cancer risks ranging from 3 × 10^−6^ to 3.07 × 10^−4^^20^, while Jalali et. al identified a carcinogenic risk of 3.45 × 10^−4^ in pathology lab employee. According to Jalali et. al, the highest mean cancer risk was among lab workers (4.44 × 10^−4^), the lowest mean cancer risk was among servants (6.16 × 10^−5^), consistent with the findings of the present study^18^.

The result of the non-cancer risk assessment is set out in Table 5. According to the results, 76.92% of the staffs were at an unacceptable level of risk (HQ > 1). The variation in the risk of non-carcinogenic effects among similar jobs in different hospitals can be attributed to the differing levels of formaldehyde exposure by staffs^17^. The lowest non-cancer risk was observed among housekeeping staff. Costa et. al suggested a potential health risk among pathology lab employees exposed to formaldehyde^36^. The result obtained from the non-cancer risk assessment indicated that the average HQ for pathologists and lab technicians was above 1. These findings are consistent with those observed in other studies^11,17^. Exposure to varying concentrations of formaldehyde, influenced by duration of exposure, building conditions, and ventilation system efficiency, leads to alterations in cancer and non-cancer risk assessment outcomes^18^. Overall, these findings suggest a correlation between extended formaldehyde exposure and an elevated risk of cancer or adverse health effects in both cancer and non-cancer risk assessments. Additionally, a noteworthy discovery is the heightened health risk faced by medical laboratory personnel, particularly those working in the pathobiology lab^37^. Studies by Jalali et al., Zhang et al., Neamtiu et al., Dang et. al reported that symptoms such as cough, burning eyes and nasal, wheezing, throat and eye irritation, asthma, etc. had high prevalence among healthcare workers exposed to formaldehyde^19,38–40^. Zhai et. al reported that coughing (11.8%), nasal irritation (39.2%), heterosmia (14.51%), and throat irritation (25.27%) were more common among the formaldehyde expose group than the non-expose group^41^. Formaldehyde exhibits high solubility in water and is rapidly absorbed by the nasal cavity, sinuses, throat, and mucous membrane of the upper respiratory tract upon exposure^18,42^. Consequently, due to the elevated potential for both carcinogenic and non-carcinogenic formaldehyde exposure among pathology staff, particularly laboratory technicians, the implementation of management measures such as strict guideline adherence and safe work protocols, along with strategies like increasing staff numbers to decrease exposure duration, as well as the adoption of engineering solutions such as localized ventilation systems and the utilization of respiratory protective equipment during sample handling and tissue processing, becomes imperative to lower the exposure levels of all employees below permissible limits. The limitations of the present study were lack of control group and not consider the effect of control systems and apply of protective equipment on the exposure rate of workers, which is suggested to be considered in future studies. In this regard, biological monitoring of occupational exposure serves as a preventive measure to evaluate chemical risks, including formaldehyde exposure. This method proves to be the most effective for assessing internal contamination, considering various exposure pathways such as respiratory, dermal, and oral routes. Factors such as the use of personal protective equipment, its efficacy, personal hygiene practices, work habits, and the quantity of formaldehyde handled at an individual level are taken into account through this approach. Biological monitoring is recommended for hazardous chemicals such as formaldehyde as the method of choice for monitoring exposure and health risk assessment. Of course, it should be noted that there is no standardized and routinely internal dose biological monitoring for formaldehyde, nevertheless, multiple studies have recommended combining environmental and biological monitoring to better understand the state of workplace^10,32,43^. Another limitation of the study is that a cross-sectional study offers a momentary glimpse into health status and, as a result, cannot unveil causal connections. In addition to this.

## Conclusion

Finally, the results of this study pointed out that according to the amount of respiratory exposure to formaldehyde, the values of carcinogenic and non-carcinogenic risk in the majority of staffs were within the definitive and unacceptable risk levels. Employing risk assessment techniques as a complementary tool in monitoring programs for respiratory exposure in the different work setting should be considered to protect the staffs against both non-cancerous and cancer-related hazards. The findings of risk assessment can be applied for management of chemical exposure and establish control measures. This procedure can significantly contribute to reducing formaldehyde exposure levels in pathology labs. Some control measures should be considered such as using proper local and dilution ventilation systems, decreasing work time, enhancing awareness and training, best work practice, making rest room with suitable sealing for staff, providing protective equipment.

## Data Availability

The datasets used and/or analyzed during the current study are available from the corresponding author on reasonable request.

## Abbreviations

ATS—DLD-78A: Thoracic society division of lung disease questionnaire
ACGIH: American conference of governmental industrial hygienists
CAS: Chemical abstract service
CDI: Chronic daily intake
CPSC: Consumer product safety commission
Ei: Exposure index
EPA: Environmental protection agency
FDA: Food and drug administration
HQ: Hazard quotient
IRIS: Integrated risk information system
IARC: International agency for research on cancer
LCR: Lifetime cancer risk
OEL: Occupational exposure limit
OSHA: Occupational safety and health administration
NRC: National research council
RFC: Reference concentration
SEGs: Similar exposure groups
TWA: Time–weight average
TLV-TWA: Time–weighted average threshold limit value
WHO: World Health Organization
